# A Comprehensive Characterization of a 10 at.% Yb:YSAG Laser Ceramic Sample

**DOI:** 10.3390/ma11050837

**Published:** 2018-05-18

**Authors:** Angela Pirri, Guido Toci, Jiang Li, Yagang Feng, Tengfei Xie, Zhaoxiang Yang, Barbara Patrizi, Matteo Vannini

**Affiliations:** 1 Istituto di Fisica Applicata “Carrara”, IFAC, Consiglio Nazionale delle Ricerche, CNR, Via Madonna del Piano 10C, 50019 Sesto Fiorentino (Fi), Italy; a.pirri@ifac.cnr.it; 2 Istituto Nazionale di Ottica, INO, Consiglio Nazionale delle Ricerche, CNR, Via Madonna del Piano 10C, 50019 Sesto Fiorentino (Fi), Italy; matteo.vannini@ino.cnr.it; 3 Key Laboratory of Transparent and Opto-functional Inorganic Materials, Shanghai Institute of Ceramics, Chinese Academy of Sciences, Shanghai 201899, China; lijiang@mail.sic.ac.cn (J.L.); fengyagang@student.sic.ac.cn (Y.F.); xietengfei120800@163.com (T.X.); yangzx@mail.sic.ac.cn (Z.Y.); 4 Istituto Nazionale di Ottica, INO, Consiglio Nazionale delle Ricerche, CNR, Via G. Moruzzi 1, 56124 Pisa, Italy; barbara.patrizi@ino.cnr.it

**Keywords:** transparent laser ceramic, ytterbium-doped laser ceramics, diode-pumped solid-state laser, tunable laser cavity

## Abstract

We report a comprehensive characterization of a 10 at.% Yb^3+^-doped YSAG (Yb:Y_3_Sc_x_Al_(5−x)_O_12_, x = 1.5) ceramic, including microstructural, spectroscopic and laser properties. Moreover, we illustrate and discuss the fabrication technique. Yb^3+^ in YSAG features a broader absorption and emission band than in traditional YAG, which is advantageous for laser applications (i.e., tunable laser sources, ultrafast pulse generation). Pumping in a quasi continuous wave regime at 936 nm, the ceramic has shown good laser performance as the maximum output power was 6.3 W with a corresponding slope efficiency (η_s_) of 67.8%. In continuous wave regime instead, the maximum output power was 5 W with η_s_ = 52.7%. The laser emission wavelengths in free running were λ_L_ = 1051 nm and λ_L_ = 1031 nm, depending on the output coupler transmission. Finally, by a tunable cavity we obtained laser emission spanning from 991.5 to 1073 nm, i.e., 81.5 nm, which is the broadest tuning range ever reported for this material, to the best of our knowledge.

## 1. Introduction

In the last years, several polycrystalline disordered materials, such as Yb:LuYAG [1,2,3] or Nd:YSAG [4], were fabricated and characterized. These gain materials show a broader emission bandwidth respect to other pure hosts, like YAG [5], Sc_2_O_3_ and Y_2_O_3;_ moreover, their emission peaks can be tuned toward longer wavelengths [6]. As a matter of fact, these laser materials are suitable for developing tunable laser systems for use in remote sensing systems as well as to generate short pulses below 100 fs [7,8]. At the same time, starting from undoped matrices with high thermal conductivity, they can preserve good thermal properties, making them suitable for high-average power lasers.

Concerning the Y_3_Sc_x_Al_(5−x)_O_12_ matrix (crystals [9,10] or ceramics), it is obtained by a partial substitution of Al^3+^ ions with Sc^3+^ ions in the Y_3_Al_5_O_12_ matrix. Sc^3+^ ion, owing a larger atomic radius respect to Al^3+^ distorts the crystal structure with a consequent increase of the lattice constant [6]. 

The modification in the crystal field results in a larger splitting of the sublevels of the upper (^2^F_5/2_) and of the lower (^2^F_7/2_) manifolds of Yb^3+^ [11], whereas the overall separation between the manifold barycenters (due to the spin-orbit splitting) remains constant [11], as it is with good approximation host-independent [12,13]. All the host-induced structural effects described above determine a red shift of the main Yb^3+^ emission peaks.

The presence of Sc^3+^ ions, in addition, produces an inhomogeneous broadening of the emission bandwidth. However, the balance between Sc^3+^ and Al^3+^ should be chosen carefully as it influences the lifetime of the laser transition and, as a consequence, the laser threshold.

The first Y_3_Sc_x_Al_(5−x)_O_12_ ceramic doped with Nd^3+^ was fabricated by Sato et al. in 2003 [14] . In 2004, Saikawa et al. [11] demonstrated the laser action of 15 at.% Yb^3+^-doped Y_3_ScAl_4_O_12_ ceramic obtained by a reactive sintering process. In particular, in Continuous Wave (CW) the laser delivered a maximum output power of 600 mW with a slope efficiency η_s_ = 72% at a lasing wavelength of λ_L_ = 1032 nm and pumping wavelength of λ_P_ = 970 nm. Pulses of 280 fs at λ_L_ = 1035.8 nm were obtained by a passively mode-locked cavity based on 5 at.% Yb:Y_3_(Sc_0.5_Al_0.5_)_5_O_12_. Moreover, it was demonstrated a tuning range of 52 nm while the slope efficiency was η_s_ = 72% [7]. In a recent paper, Ma and co-workers [15] have tested a Yb:Y_3_ScAl_4_O_12_ ceramic obtaining mode-locked laser pulses with a duration of 96 fs at 1052 nm, and a repetition rate of 102 MHz; they have obtained a maximum average output power that was 51 mW.

This paper is devoted to exploring the overall potentiality of a laser prototype based on a 10 at.% Yb:Y_3_Sc_1.5_Al_3.5_O_12_ ceramic. The sample has been fabricated by solid-state reaction combined with vacuum sintering and its microstructure has been characterized through Field Emission Scanning Electron Microscopy (FESEM) and X-ray Diffraction (XRD). We present a spectroscopic characterization of the ceramic, in particular, the absorption and the emission cross section spectra and the upper laser level lifetime. Laser tests were carried out in Quasi-Continuous Wave (QCW) and in CW operation modes at room temperature. The implementation of a tunable cavity has allowed measuring a tuning curve as wide as 81.5 nm, which is comparable with data obtained with Yb^3+^ in fluoride hosts [16,17,18]. To the best of our knowledge, this is the broadest tuning range reported in literature for this material.

## 2. Ceramic Fabrication Technique

Commercial powders of Y_2_O_3_ (99.99%, Yuelong, Shanghai, China), Sc_2_O_3_ (99.99%, Jingyun, Shanghai, China), α-Al_2_O_3_ (99.99%, Fenghe, Shanghai, China), and Yb_2_O_3_ (99.99%, Zhongkai, Shandong, China) were used as staring materials to fabricate 10 at.% Yb:Y_3_Sc_1.5_Al_3.5_O_12_ (i.e., Yb_0.3_Y_2.7_Sc_1.5_Al_3.5_O_12_) ceramics. MgO powder (99.998%, Alfa Aesar, Tianjin, China) and tetraethoxysilane (TEOS, >99.999%, Alfa Aesar, Tianjin, China) were used as sintering aids. The powders were mixed in ethanol and ball-milled with high-purity corundum balls for 12 h. After the ball milling, the slurry was dried at 70 °C for 2 h in an oven and then sieved through 200-mesh screen.

The powder mixtures were calcined at 600 °C for 4 h to remove the organic components. The calcined powders were uniaxially pressed into 18 mm diameter pellets at low pressure (46 MPa) and then the green bodies obtained were isostatically pressed at 250 MPa at room temperature. The pellets were sintered at 1820 °C for 30 h in a tungsten mesh-heated vacuum furnace and then annealed in air at 1450 °C for 20 h to remove the oxygen vacancies. Finally, the ceramics were mirror-polished on both surfaces and reduced to 2.0 mm of thickness for optical measurements. The polished specimens were thermally etched at 1450 °C for 3 h to expose the grain boundaries for the microstructural analysis.

**Figure 1:**
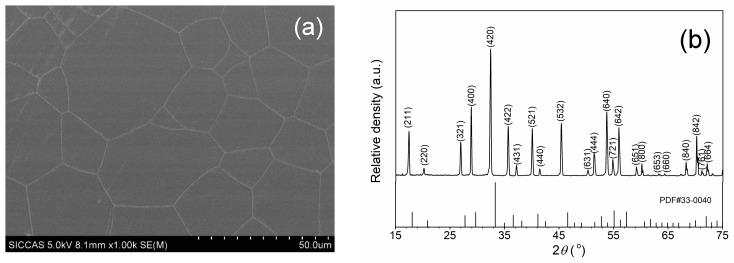
(**a**) Field Emission Scanning Electron Microscopy FESEM micrograph of the thermally etched surface of a 10 at.% Yb:Y_3_Sc_1.5_Al_3.5_O_12_ transparent ceramics sintered at 1820 °C for 30 h; (**b**) XRD pattern of the sample.

Figure 1a shows the FESEM micrograph of the thermally etched surface of a 10 at.% Yb:Y_3_Sc_1.5_Al_3.5_O_12_ transparent ceramics sintered at 1820 °C for 30 h. The grain composing the sample have an average size of 44 μm, determined by the linear intercept method (more than 200 grains were counted); the grain boundaries are very clean; at the grain boundaries and in the inner grains the presence of pores is quite negligible.

Figure 1b shows the XRD pattern of the Yb:YSAG sample. The ceramics crystal structure shows the expected cubic garnet phase with a lattice parameter of 1.2225 nm, which is about 2% larger than that of the lattice constant reported for 10 at.% Yb^3+^ doped YAG that is 1.200058 nm [19].

## 3. Spectroscopic and Optical Characterization

The spectroscopic characterization was aimed mainly to determine the absorption and the emission cross section spectra of the Yb^3+^ in the sample, and to determine the upper laser level lifetime.

**Figure 2:**
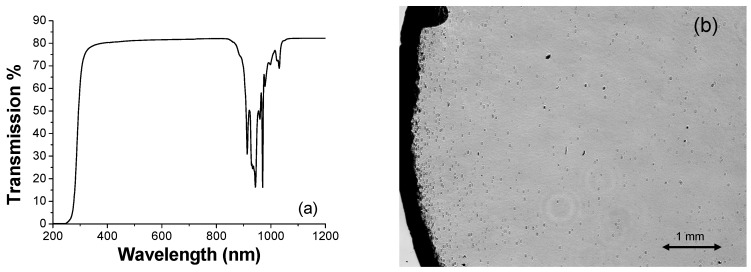
(**a**) Transmission spectrum of the 1.85 mm-length ceramic sample under test; (**b**) Microscopic transmission image of the sample. The size of the displayed area is ∼5.9 × 4.4 mm. The dark band on the left side is the edge of the sample.

The transmission spectrum of the ceramic sample was measured with a Perkin Elmer Lambda 1050 spectrophotometer, with a spectral resolution of 1 nm (Figure 2a).

The transmission spectrum, see Figure 2a, clearly shows the absorption bands due to the Yb^3+ 2^F_5/2_–^2^F_7/2_ transition in the range 900–1020 nm. No other absorption features can be seen between 300 and 900 nm.

Regarding the theoretical transmission, to our knowledge no refractive index data are available in literature for this specific composition; Allik et al. [10] reported a value of the refractive index of 1.873 at 632.8 nm for Nd-doped Y_3_Sc_2_Al_3_O_12_, from which a theoretical transmission value of 82.39% (due to the Fresnel reflections) can be calculated. This is very similar to the measured value of 81.65% obtained at the same wavelength. The scattering coefficient at 632.8 nm is then about 0.05 cm^−1^.

Figure 2b reports the microscopic optical image (in transmission) of the sample, showing the distribution of the residual pores that appear as dark spots in the image. The optical uniformity of the sample is quite good, and the residual pores are mainly distributed near to the edge of the sample. Their presence does not influence significantly the transmission of the sample (see Figure 2a). 

**Figure 3:**
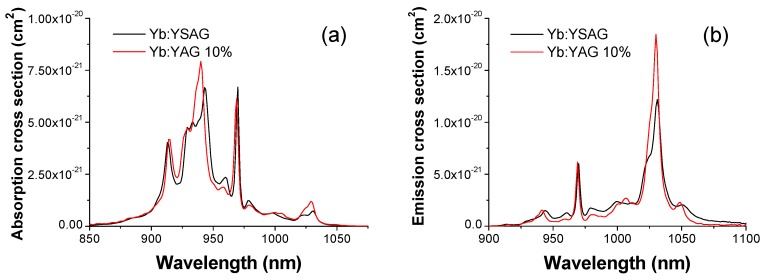
(**a**) Absorption cross section spectrum (*σ_abs_*) for the 10 at.% Yb:Y_3_Sc_1.5_Al_3.5_O_12_ (Yb:YSAG) sample, obtained from the transmission spectrum reported in Figure 2a. For comparison it is also shown the absorption cross section spectrum of a 10 at.%-doped Yb:YAG ceramics produced with the same method; (**b**) Emission cross section spectra (*σ_em_*) for Yb:YSAG and Yb:YAG calculated from the absorption cross section spectra using the reciprocity method (see text).

The cations sites density of the Y_3_Sc_1.5_Al_3.5_O_12_, calculated from the lattice constant value of 1.2225 nm (determined by X-ray diffraction measurements), is 1.314 × 10^22^ ions/cm^3^. From the transmission spectrum of Figure 2a it was then possible to calculate the absorption cross section spectrum, shown in Figure 3a.

The absorption cross section of Yb^3+^ features a broad peak at about 943 nm, whereas the zero-phonon line peak is located at 969.7 nm. By comparing it with the absorption cross section spectrum of Yb:YAG (also shown in Figure 3a), it appears that both the main absorption peak (peaked at 939.4 nm in YAG) and the zero line peak (at 968.93 nm in YAG) are slightly shifted toward longer wavelengths. Moreover, at about 1030 nm (one of the main lasing wavelengths) the ground level absorption cross section of Yb^3+^ in YSAG is lower than in YAG.

The emission cross section spectrum *σ_em_*(*λ*) (Figure 3b) was calculated from the absorption cross section spectrum *σ_ab_*(*λ*) by means of the reciprocity (McCumber) formula [20]:

(1) $$\sigma_{em}\left(\lambda \right)=\sigma_{ab}\left(\lambda \right)\left[Z_{l}/Z_{u}\right]\exp\left[\left(E_{ZL}-hc/\lambda \right)/k_{B}T\right]$$

where *E_ZL_* is the energy of the zero-phonon line transition, *h* is the Planck’s constant, *c* is the speed of light, *k_B_* is the Boltzmann constant, *T* is the temperature, *Z_l_* and *Z_u_* are the partition functions of the lower and upper level, respectively. For the evaluation of *Z_l_*/*Z_u_* we used the values of the energy levels for Yb in YSAG and YAG reported by Saikawa et al. [11]. Even though these values are valid for the host composition Y_3_Sc_2_Al_3_O_12_, the resulting value of *Z_l_*/*Z_u_* at room temperature for Y_3_Sc_2_Al_3_O_12_ (0.8835) is very close to that of YAG (0.8817). We assumed then that in Equation (1) the same value can be used as a good approximation for the value of *Z_l_*/*Z_u_* in Y_3_Sc_1.5_Al_3.5_O_12_.

These measurements confirm the trend already observed by Saikawa et al. [11]: the main emission cross section peak in Yb:Y_3_Sc_1.5_Al_3.5_O_12_, located at about 1031 nm, has a lower peak value (about 1.2 × 10^−21^ cm^2^) than the corresponding peak in Yb:YAG (about 1.8 × 10^−21^ cm^2^ at 1030 nm), but it is significantly broader than in YAG (13 nm vs. 8.5 nm FWHM). Furthermore, the emission spectrum of Yb:Y_3_Sc_1.5_Al_3.5_O_12_ shows a relatively high tail extending up to 1075 nm and above, whereas in the same spectral region the emission cross section of Yb:YAG decreases quite rapidly. It must be noticed that for a crystal with composition Yb:Y_3_Sc_2_Al_3_O_12_ Dong et al. [9] have reported a peak emission cross section of about 1.9 × 10^−20^ cm^2^ at 1030.7 nm (that is larger than both our value and that reported by Saikawa et al. [11]) and a slightly larger peak bandwidth (∼13.3 nm FWHM).

The lifetime of the upper level was measured by means of the so-called pinhole method to compensate for radiation trapping effects [21,22,23] and using a pulsed ns Ti:Sapphire laser as excitation source. We obtained a lifetime value of 966 ± 6 μsec. This is comparable with the value commonly accepted for Yb:YAG (∼950 μsec, [23]), but shorter than the value (∼1.1 msec) reported for both the ceramics with composition Yb:Y_3_ScAl_4_O_12_ (Saikawa et al. [11]) and for the crystal with composition Yb:Y_3_Sc_2_Al_3_O_12_ (Dong et al. [9]). The shortening of the lifetime could be related to some non-radiative decay processes affecting the upper laser level of Yb^3+^, due to defects in the crystal lattice. On the other hand, it must be noticed that in Ref. [11] the upper level lifetime was not measured directly, but determined as a fitting parameter to match the emission spectrum obtained from the reciprocity formula and from the Füchtbauer–Ladenburg method applied to the fluorescence spectrum. Ref. [9], instead, actually reports the measured fluorescence lifetime, which could be affected by radiation trapping effects, resulting in a larger value than the actual upper level lifetime.

## 4. Laser Results and Analysis

**Figure 4:**
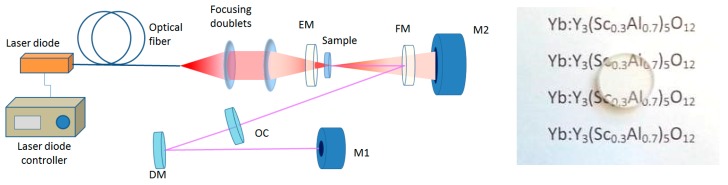
V-shaped laser cavity with a folding half angle of 10°. C: Ceramic sample; EM: End-Mirror; FM: Folding-Mirror (ROC 100 mm); OC: Flat Output Coupler mirror; M1 and M2: Power Meters, DM: Dichroic Mirror. The distance between FM and EM is 56 mm, and the distance between FM and OC is 220 mm. the ceramic was pumped at 936 nm. The inset on the right shows the sample used in the experiment (∅ 13.5 mm).

The laser behavior of the ceramic was tested by using the V-shape cavity reported in Figure 4. The uncoated ceramic, of 1.85 mm length and doping concentration of 10 at.% Yb^3+^, was welded by a sheet of indium on a copper heat-sink and cooled with water at 17 °C. The input and output facets of the sample were polished for optical measurements.

The ceramic was pumped by a fiber-coupled laser-diode which emits at 936 nm with a Gaussian pump intensity distribution in the region of the focal plane (i.e., waist radius around 95 µm at 1/e^2^, measured with a Charge-Coupled Device (CCD) camera). With this cavity we performed two sets of measurements. In the first, the ceramic was pumped in QCW regime, at 10 Hz of repetition rate with a duty factor of DF = 20%, in order to limit the thermal load into the ceramic. In the second set of measurements, the ceramic was pumped in CW regime. With both schemes the maximum incident pump power was 19.2 W.

**Figure 5:**
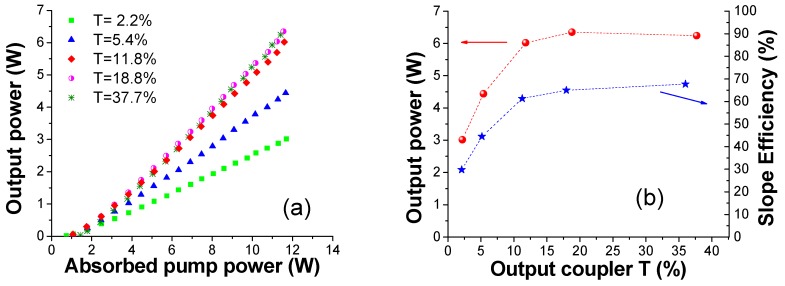
QCW laser output power versus the absorbed pump power (**a**). Maximum output power and the corresponding slope efficiency as a function of the OCs transmission, T (%) (**b**). The unsaturated absorptions of the ceramic are 68.2% (QCW) and 73.8% (CW), respectively.

Figure 5 reports the QCW laser output power as a function of the absorbed pump power, P_abs_, measured by using different output coupler mirrors; their transmission ranged from *T* = 2.2% to *T* = 37.7%. The highest slope efficiency was achieved with *T* = 18.8%, i.e., η_s_ = 67.8%, while the laser maximum output power of 6.3 W at λ_L_ ∼ 1031 nm was obtained with both the output coupler mirrors with *T* = 18.8% and with *T* = 37.7%. The longest wavelength in free running lasing, i.e., λ_L_ = 1051 nm, is measured with *T* = 2.2% (which has also given the lowest laser threshold, i.e., P_th_ = 0.74 W).

Concerning the red shift of the emission wavelength, observed using an Output Coupler (OC) with a lower transmittance, this effect can be addressed to the quasi three-level system behavior of the Yb^3+^. As a matter of fact, using OCs with higher transmission, the cavity losses increases, and in turn, the fraction of the excited population needed to reach the lasing threshold increases as well determining a shift of the peak of the effective gain spectrum toward shorter wavelengths (gain-tuning effect [8]).

From the values of the slope efficiency obtained with output couplers with different transmission, it was possible to calculate the overall internal losses of the cavity by means of the Caird formula [24]. We obtained a single pass loss of 1.7%, which is slightly higher than the estimated internal transmission losses of the sample (∼0.7%) and is probably also affected by the residual losses due to the Fresnel reflection at the interfaces.

**Figure 6:**
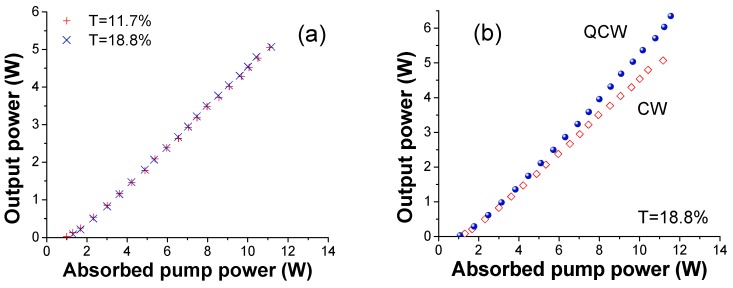
CW laser output power (P_out_) versus the absorbed pump power (P_abs_) (**a**) with two OCs, i.e., T = 11.7% and T = 18.8%; In (**b**) are reported the data obtained with T = 18.8% in QCW and CW.

Remarkable results are confirmed in CW pumping. Two different measurements were performed using mirrors with *T* = 11.7% and *T* = 18.8% as output couplers. Very similar results in terms of output power and slope efficiency were found with the two output couplers as reported in Figure 6a. In particular, the laser delivered P_out_ = 5 W with both output couplers while the slope efficiency were η_s_ = 52.2% and η_s_ = 52.7%, respectively; the laser threshold and the emission wavelength remained instead unchanged. It must be noticed that in CW the pump absorption is slightly increased as a consequence of the emission wavelength red shift of the pump laser. From these data it appears that the thermal load determines only a modest decrease of the laser performance, in particular, the maximum output power decreases from 6 W (η_s_ = 65.1%) to 5 W (η_s_ = 52.7%) switching from QCW to CW at the same pump power values (Figure 5b). These results demonstrate the high thermal quality of this material. Finally, we note that the laser threshold and the emission wavelength remained unchanged, i.e., P_th_ ∼ 1 W, λ_L_ ∼ 1031 nm.

**Figure 7:**
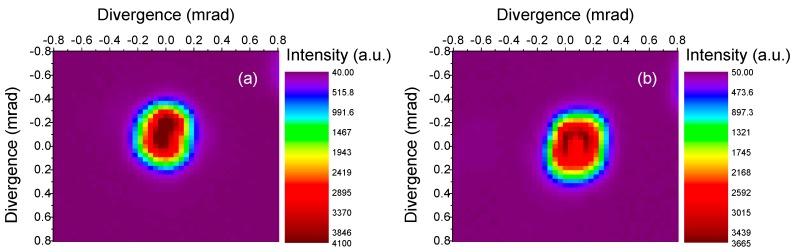
Far-field intensity distribution of the laser beam in QCW (**a**) and CW (**b**) pumping conditions.

Regarding the beam quality structure, in the near field the laser beam has about a Gaussian intensity distribution. The beam quality factor M^2^ was measured by means of a CCD camera (model BC106, Thorlabs Inc., Newton, NJ, USA) and a beam analysis software, using the output coupler with transmittance 18.8% (that provided the highest output power both in QCW and in CW pumping conditions). The measurement was carried out at 3.3 W of output power (i.e., about half of the maximum output power) and it resulted in M^2^_x_ = 2.2, M^2^_y_ = 2.4 in QCW and M^2^_x_ = 2.5, M^2^_y_ = 2.8 in CW pumping conditions (here, *x* and *y* refer to the directions respectively parallel and perpendicular to the cavity folding plane, see Figure 4). The far-field intensity distribution both in QCW and in CW pumping mode is shown in Figure 7. The beam has then a multimode structure, because the pump beam in the sample (radius at 1/e^2^ 95 μm, see above) is larger than the cavity TEM_00_ mode radius (about 48 μm) in the pumped volume. The asymmetry between the *x* and the *y* direction is due to the slight cavity astigmatism introduced by the tilted spherical mirror (i.e., the Folding Mirror FM, see Figure 4). In any case, it must be noticed that no attempt was carried out to optimize the beam quality, but only the power extraction.

**Figure 8:**
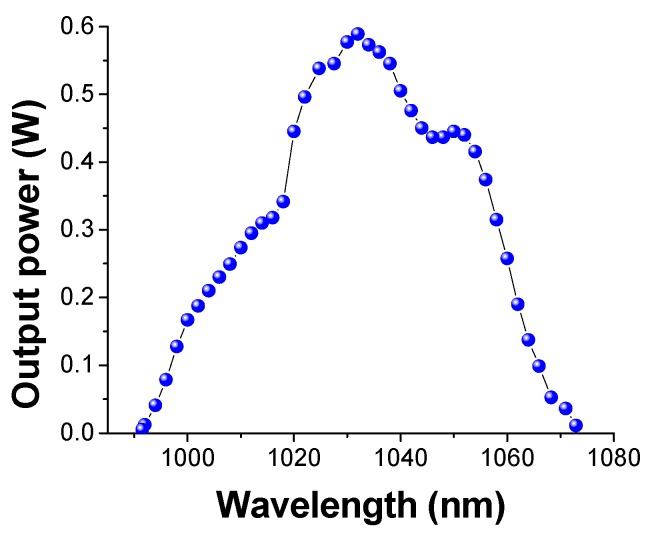
QCW tuning curve. The injected pump power was 19.2 W corresponding to P_abs_ = 11.7 W.

The tunability range of the ceramic was explored by inserting a ZnSe prism in Brewster configuration (apex angle 41.8°) between the FM and the OC. The emission wavelength was measured by a spectrometer with a focal length of 60 cm equipped with a multichannel detector. The spectral resolution was 0.4 nm. We have measured the output power at several wavelengths (see Figure 8), obtaining a tuning range as wide as 81.5 nm (i.e., from λ_L_ = 991.5 nm to λ_L_ = 1073 nm). The curve shows two main peaks at λ_L_ = 1030 nm and λ_L_ = 1050 nm with an output power of 118 mW and 89 mW, respectively. The laser emission line width is about 2.5 nm across the whole tuning range. It is worth noting that the tuning limit at short wavelengths is also affected by the cutoff of the transmission curve of the End Mirror EM (see Figure 4).

## 5. Conclusions

In this experiment, we have investigated the microstructural and spectroscopic properties as well as the laser performance of a 10 at.% Yb:YSAG ceramic fabricated by reactive vacuum sintering. Concerning the spectroscopic characterization, we have determined the absorption and emission cross sections and the lifetime of the upper state of the laser transition. The mixed composition has shown a significant broadening of the main peak of the emission spectrum near 1031 nm with respect to YAG (13 nm vs. 8.5 nm, FWHM). By comparing our results with previous literature on Yb:YSAG [7,9], it appears that the general trend is toward a broadening of the emission spectrum for increasing content of Sc^3+^, as discussed above. The upper level lifetime resulted slightly longer than that reported for Yb:YAG, but shorter than those reported for the ceramics with composition Yb:Y_3_ScAl_4_O_12_ (Saikawa et al. [11]) and for the crystal with composition Yb:Y_3_Sc_2_Al_3_O_12_ (Dong et al. [9]). The discrepancies in the upper level lifetime could be due to the use of different assessment methodologies, as pointed out above.

Good results are achieved in terms of laser performance such as output power, slope efficiency and laser threshold. A direct comparison can be made with the CW emission results reported by Saikawa et al. [7,11]. The authors obtained a maximum slope efficiency η_s_ of 72% (maximum output power ∼0.6 W) under pumping at 970 nm and a maximum η_s_ of 54% (max. output power ∼0.8 W) pumping at 940 nm, using a CW Ti:Sapphire laser as a pump source. The values of the slope efficiencies here reported (up to 67.7%) are comparable with the previous results, but the output power levels reached in our experiments are much higher than those previously reported in [5,7,11]. It is important to emphasize that our experiments have been carried out using a high power diode laser as a pump source, which is less favorable in terms of beam quality with respect to the Ti:Sapphire laser used in [7,11], but much more relevant in view of practical applications. Finally, the observed tuning range (from λ_L_ = 991.5 nm to λ_L_ = 1073 nm) is much broader than previously reported (from about 1015 nm to 1065 nm) [7].

The results reported here demonstrate a significant advancement in the quality of Yb:YSAG ceramics as a laser gain material. The improvement in the laser performance, indeed, is favored by the high optical and spectroscopic quality of the sample. Further investigations will address the comparison of the spectroscopic and laser performances of Yb:YSAG ceramics with different Sc/Al balance, in order to obtain additional improvements.
